# Quantification of liver fibrosis: extracellular volume fraction using an MRI bolus-only technique in a rat animal model

**DOI:** 10.1186/s41747-019-0100-y

**Published:** 2019-05-29

**Authors:** Julian A. Luetkens, Sabine Klein, Frank Träber, Wolfgang Block, Frederic C. Schmeel, Alois M. Sprinkart, Daniel L. R. Kuetting, Frank E. Uschner, Robert Schierwagen, Daniel Thomas, Jonel Trebicka, Guido M. Kukuk

**Affiliations:** 1 Department of Radiology, University Hospital Bonn, Venusberg-Campus 1, 53127 Bonn, Germany; 2 Department of Internal Medicine I, University Hospital Bonn, Venusberg-Campus 1, 53127 Bonn, Germany; 3 European Foundation for the Study of Chronic Liver Failure, Barcelona, Spain; 4 Faculty of Health Sciences, University of Southern Denmark, Odense, Denmark; 5 Institute for Bioengineering of Catalonia, Barcelona, Spain

**Keywords:** Extracellular space, Fibrosis, Liver, Magnetic resonance imaging, Rats (Sprague-Dawley)

## Abstract

**Background:**

To determine the utility of single-contrast-bolus hepatic extracellular volume (ECV) fraction measurement at different time points to detect and quantify hepatic fibrosis.

**Methods:**

Different grades of liver fibrosis were induced in 23 male Sprague-Dawley rats by carbon-tetrachloride (CCl_4_) intoxication. In ten control rats, no fibrosis was induced. Native T1 values and ECV fraction were assessed by using quantitative magnetic resonance imaging (MRI) mapping; only one contrast bolus was applied (gadobutrol 0.1 mmol/kg). ECV values were determined 5, 15, and 25 min after injection. Hepatic fibrosis was quantified histologically by Sirius red staining.

**Results:**

For the 8-week-CCl_4_ group, the ECV fraction values obtained 5 (23.5 ± 4.8%, mean ± standard deviation), 15 (23.6 ± 4.8%), and 25 min (23.7 ± 4.7%) after injection were constant over time (*p* = 0.998); constant data 5–25 min after injection were also observed for the 16-week-CCl_4_ group and controls. Liver ECV after 15 min significantly increased with the severity of fibrosis: 18.0 ± 3.0% (controls) *versus* 23.6 ± 4.8% (8-week-CCl4) *versus* 30.5 ± 3.3% (16-week-CCl4) (*p* <  0.001). ECV values after 5, 15, and 25 min significantly correlated with Sirius red staining (*p* <  0.001 for all parameters).

**Conclusions:**

Hepatic ECV obtained using a single-contrast-bolus technique can be measured 5, 15, and 25 min after injection, obtaining constant values over time, each of them being suitable to detect diffuse hepatic fibrosis. In clinical practice, post-contrast T1 relaxation times for liver ECV fraction determination might be obtained at only one time point.

## Key points

- Extracellular volume fraction was increased in liver fibrosis in an experimental animal model.
- Single-contrast-bolus extracellular volume fraction measurements of the liver were constant over time (from 5 to 25 min).
- A single post-contrast T1 relaxation time measurement could be sufficient for extracellular volume fraction assessment in clinical practice.

## Background

Chronic liver disease has become a major public health concern in Western populations [1]. Any chronic liver injury may lead to fibrosis, which distorts normal liver architecture by the expansion of the extracellular space, and impairs hepatic function [2]. The development of liver fibrosis is an unfavourable sign, which is tightly linked to progression of liver disease, portal hypertension and hepatocellular carcinoma [3]. Since the development of fibrosis increases the risk for progression towards cirrhosis and hepatocellular carcinoma, the presence of fibrosis requires interventions (*e.g.*, life style modifications in non-alcoholic steatohepatitis, alcohol cessation in alcohol-related liver disease, suppression of immune response in autoimmune hepatitis, or antiviral agents in hepatitis C infection [4]). Therefore, timely and correct diagnosis and accurate staging of liver fibrosis immediately affect prognosis and patient management.

Besides liver biopsy with its known drawbacks such as risk of severe complications and a high intra- and inter-observer variability [5], non-invasive techniques such as transient elastography are increasingly preferred in order to grade liver fibrosis. Almost all non-invasive techniques require additional expensive devices and trained personnel [6]. On the other hand, in almost all patients with chronic liver disease, there is a clinical need for imaging. Among other imaging modalities (*e.g.*, dynamic computed tomography and contrast-enhanced ultrasound), contrast-enhanced magnetic resonance imaging (MRI) is the reference standard for liver imaging and exclusion of malignancies such as hepatocellular carcinomas [7].

Very recently, MRI-derived extracellular volume (ECV) fraction using T1 mapping techniques was described as a new tool for the non-invasive assessment of liver fibrosis [8]. ECV values are calculated from the change in relaxation rate (R1 = 1/T1) of blood and liver parenchyma corrected for the haematocrit [9, 10]. The ECV is postulated to be constant over time under equilibrium conditions [11]. This equilibrium can be established by the administration of a primed slow intravenous contrast infusion [9]. However, the initially described technique is time-consuming and thus not routinely applicable in clinical practice. A more feasible approach is the use of a bolus-only technique, which assumes that after some time after a single contrast bolus, a dynamic equilibrium can be achieved [12, 13], which would facilitate equivalent ECV measurement.

The purpose of this study was to assess the stability of liver ECV determination over time using the more simplified bolus only technique and to evaluate the influence of different time points of ECV measurements for the evaluation of liver fibrosis in an animal model of fibrosis.

## Methods

The responsible committee for animal studies of German federal state North Rhine-Westphalia approved the study (LANUV: 84–02.04.2014.A137). All experiments were performed in accordance with relevant guidelines and regulations. A part of the animal studies has been reported in a previous proof-of-concept study [10].

### Animal models of cirrhosis

A total of 33 male Sprague-Dawley rats were used for our experiments. In 23 rats, liver fibrosis was induced using a toxic model of liver cirrhosis. Rats regularly underwent exposure to carbon tetrachloride (CCl_4_) inhalation of 2 L/min as described previously [14]. This procedure results in micronodular cirrhosis with portal hypertension after 14–16 weeks. Rats underwent exposure to CCl_4_ for 8 (*n* = 10) and 16 weeks (*n* = 13). Untreated age-matched rats were used as controls (*n* = 10) and did not receive CCl_4_.

### MRI protocol

All scans were performed on a 3-T whole-body MRI scanner (Ingenia 3 T, Philips Healthcare, Best, The Netherlands) using an eight-channel small extremity coil for signal reception. Rats were anaesthetised for MRI scans by intramuscular injection of ketamine/xylazine (78 mg/kg and 10 mg/kg body weight). For the determination of pre- and post-contrast T1 relaxation times, high-resolution T1 maps were acquired. T1 maps were acquired before and 5, 15, and 25 min after contrast injection (0.1 mmol/ kg of body weight of gadobutrol, Gadovist, Bayer Healthcare, Leverkusen, Germany). For unenhanced hepatic T1 mapping, a 4(10)10 modified Look-Locker inversion-recovery (MOLLI) acquisition scheme was applied, as previously described [10], with the following technical parameters: time of repetition 4.7 ms, time of echo 2.3 ms, flip angle 20°, parallel imaging factor 1.5, acquired voxel size 0.8 × 0.8 × 1.5 mm, reconstructed voxel size 0.25 × 0.25 × 1.5 mm, scan duration 1 min 32 s. Contrast-enhanced T1 mapping was performed with a 3(6)2(6)8 MOLLI scheme (scan duration 01 min 44 s).

### Haematocrit and haemodynamic measurements

After the scan, median laparotomy was performed and a polyethylene-50 catheter was introduced into an ileocecal vein and advanced to the portal vein for the measurement of portal pressure [15]. The left femoral artery was cannulated with a polyethylene-50 catheter for measurement of the mean arterial pressure and blood withdrawal. Blood haematocrit levels were assessed by centrifugation of blood samples. All rats were sacrificed shortly after the MRI scans.

### Sirius red staining

For Sirius red staining always the right lobe was used. Three parts of the right lobe were paraffin-embedded and stained. The average amount of all three stained areas (%) was taken for further quantification. For the detection of collagen fibres, liver specimens were fixed in 10% formalin, paraffin-embedded, and stained in 0.1% Sirius red in saturated picric acid (Chroma, Munster, Germany) using methods as previously described [15].

### Image analysis

**Fig. 1:**
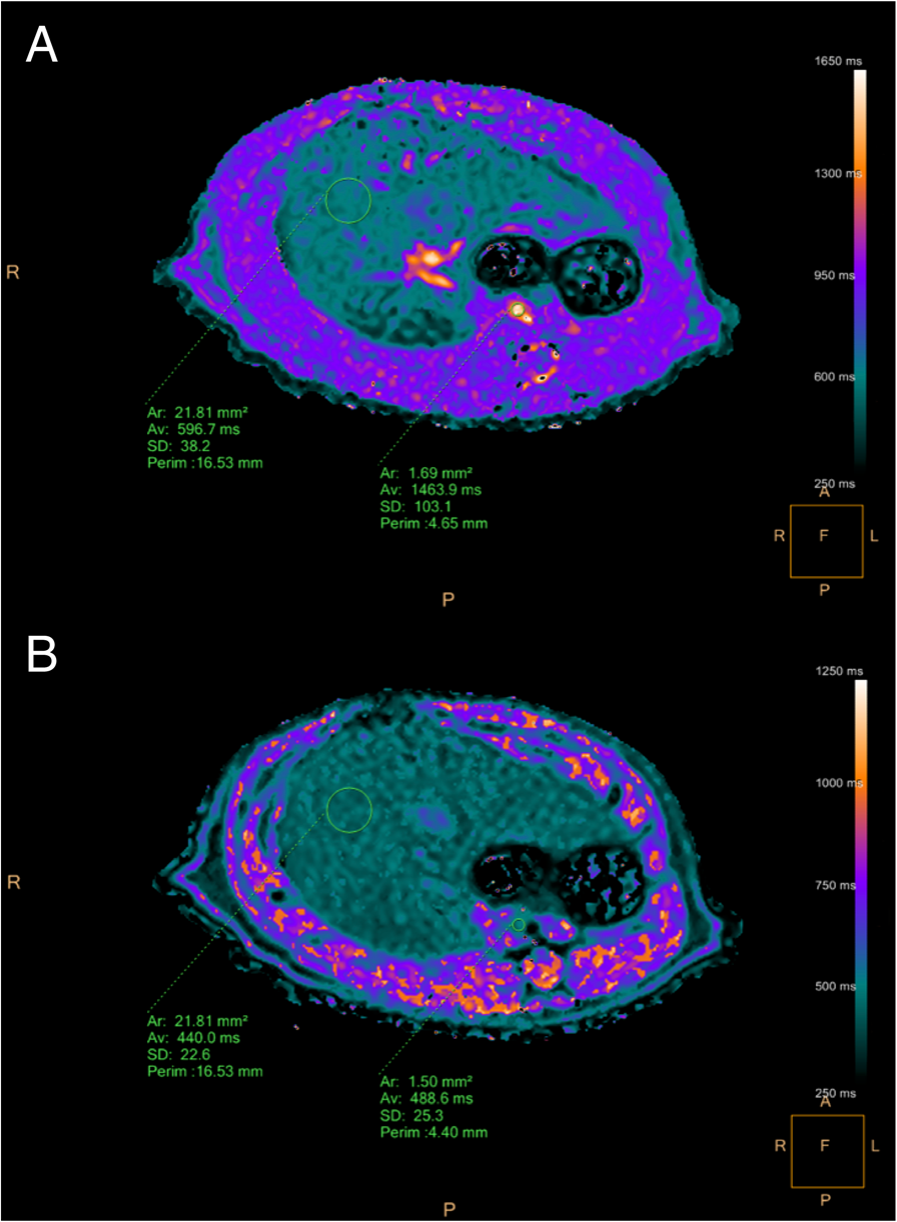
Exemplary measurements of hepatic T1 relaxation times. Circular regions of interest are drawn in the right lobe of the liver and in the abdominal aorta. Measurements are given for pre-contrast (**A**) and 15 min post-contrast images (**B**)

Hepatic T1 relaxation times were extracted from relaxation maps by physicians blinded to the stages of fibrosis and time of image acquisition, using software (Philips IntelliSpace Portal 8.0, Best, The Netherlands). For each rat, a single representative region of interest (ROI) excluding hepatic vasculature was drawn in the central right lobe of the liver. For the same animal, the same ROI was placed accordingly on all other relaxation maps (see Fig. 1). For each ROI, mean T1 values were recorded and used for final analysis. T1 values of the blood pool were obtained from the abdominal aorta on the transversal maps. ECV values were normalised for haematocrit and calculated from pre- and post-contrast T1 values using the following equation [16]:

$$ \mathrm{ECV}=\frac{\left(1/\mathrm{T}1\ \mathrm{liver}\ \mathrm{post}-\mathrm{contrast}-1/\mathrm{T}1\ \mathrm{liver}\ \mathrm{pre}-\mathrm{contrast}\right)}{\left(1/\mathrm{T}1\ \mathrm{blood}\ \mathrm{post}-\mathrm{contrast}-1/\mathrm{T}1\ \mathrm{blood}\ \mathrm{pre}-\mathrm{contrast}\right)}\times \left(1-\mathrm{haematocrit}\right) $$

### Statistical analysis

Statistical analysis was performed using Prism 7 (GraphPad Software, Inc., La Jolla, CA, USA) and IBM SPSS Statistics 23 (IBM Corporation, Armonk, NY, USA). Descriptive data were presented as mean ± standard deviation. For multiple comparisons, the Kruskal-Wallis test followed by Dunn’s multiple comparisons test was used. The level of statistical significance was set to *p* <  0.05. Correlation analysis was performed using Spearman’s rank correlation coefficient.

## Results

**Fig. 2:**
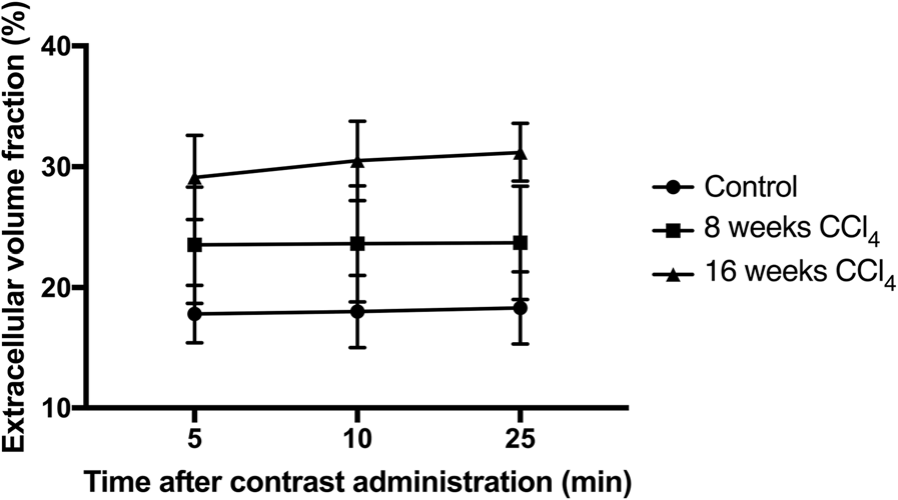
Change on mean extracellular volume (ECV) fraction measurements over time. Measurements are given for the control group and for the 8 and 16 weeks carbon-tetrachloride (CCl_4_) groups

The mean ECV in the control group was 17.8 ± 2.4% at 5 min, 18.0 ± 3.0% at 15 min, and 18.3 ± 3.0% at 25 min (*p* = 0.910); in the 4-week CCl4 group, 23.5 ± 4.8%, 23.6 ± 4.8%, and 23.7 ± 4.7%, respectively (*p* = 0.998); in the 16-week CCl4 group, 29.1 ± 3.5%, 30.5 ± 3.3%, and 31.2 ± 2.4%, respectively (*p* = 0.261). It increased only slightly over time without reaching statistical significance (Fig. 2).

We observed a continuous increase in the percentage of positive staining for Sirius red for the different severities of liver fibrosis (0.1 ± 0.1% in controls *versus* 3.3 ± 2.6% in 8-week CCl_4_ group *versus* 25.1 ± 3.2% in 16-week CCl_4_ group (*p* < 0.001). Likewise, mean native T1 values increased with different severities of liver fibrosis: 593.3 ± 10.3 ms in controls *versus* 625.5 ± 38.3 ms in the 8-week CCl_4_ group *versus* 645.6 ± 41.0 ms in the 16-week CCl_4_ group (*p* = 0.007).

The mean ECV acquired 15 min after contrast administration increased with different severities of liver fibrosis: 18.0 ± 3.0% in controls *versus* 23.6 ± 4.8% in the 8-week CCl_4_ group *versus* 30.5 ± 3.3 ms in the 16-week CCl_4_ group (*p* < 0.001) (Table 1).

**Table 1:** General characteristic of different groups of CCL_4_ rats

Parameter	Control (*n* = 10)	8-week CCl_4_ (*n* = 10)	16-week CCL_4_ (*n* = 13)	*p* value
Body weight (g)	488.0 ± 81.1	368.7 ± 45.7*	499.3 ± 54.8^#^	< 0.001
Liver weight (g)	15.3 ± 2.7	14.6 ± 2.3	17.2 ± 2.8	0.081
Mean arterial pressure (mmHg)	113.4 ± 16.4	103.9 ± 22.7	102.5 ± 21.4	0.287
Portal pressure (mmHg)	9.0 ± 1.3	15.0 ± 2.6*	19.1 ± 7.5*	< 0.001
Positive staining Sirius red (%)	0.1 ± 0.1	3.3 ± 2.6*	25.1 ± 3.2*^#^	< 0.001
Native T1 relaxation time (ms)	593.3 ± 10.3	625.5 ± 38.3	645.6 ± 41.0*	0.007
5 min extracellular volume fraction (%)	17.8 ± 2.4	23.5 ± 4.8	29.1 ± 3.5*	< 0.001
15 min extracellular volume fraction (%)	18.0 ± 3.0	23.6 ± 4.8	30.5 ± 3.3*^#^	< 0.001
25 min extracellular volume fraction (%)	18.3 ± 3.0	23.7 ± 4.7	31.2 ± 2.4*^#^	< 0.001

Data are presented as mean ± standard deviation. *CCl*_*4*_ carbon tetrachloride. * *p* < 0.05 *versus* control; # *p* < 0.*05* *versus* 8-week CCl_4_

**Fig. 3:**
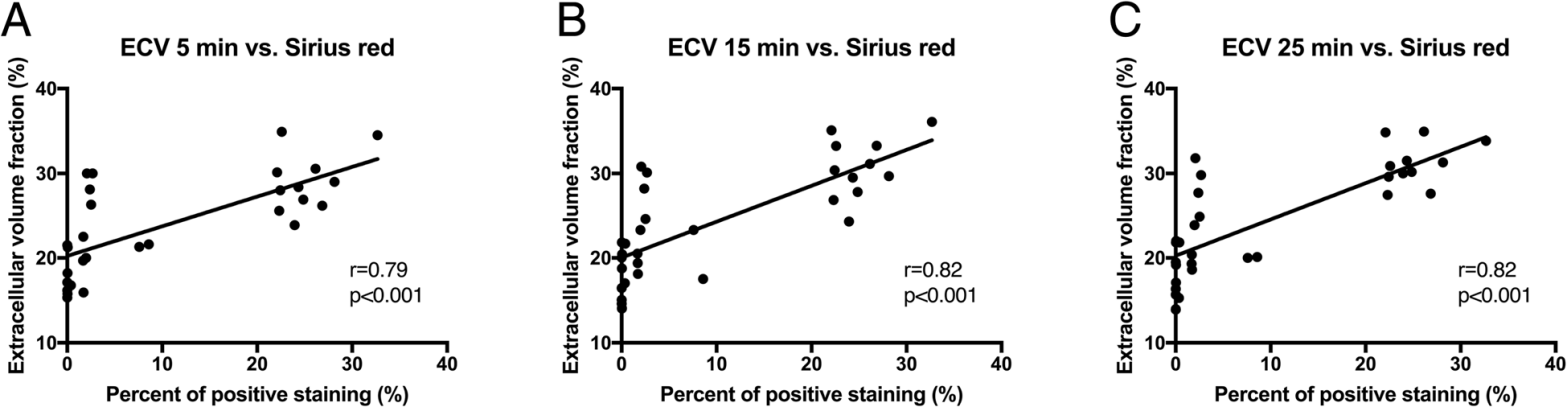
Scatter plots showing correlations between extracellular volume (ECV) fraction and positive staining for Sirius red 5 (**A**), 15 (**B**), and 25 min (**C**) after contrast administration

ECV values at 5, 15, and 25 min were all significantly correlated with hepatic Sirius red staining (*p* < 0.001 for all correlations; Fig. 3). Also, ECV values at 5 min (*r* = 0.41; *p* = 0.027), at 15 min (*r* = 0.39; *p* = 0.029), and at 25 min (*r* = 0.48; *p* = 0.006) were slightly correlated with portal vein pressure.

## Discussion

This prospective study demonstrated that a simplified one bolus-only MRI technique for quantifying the hepatic ECV is accurate to diagnose and grade fibrosis in an animal model. This is based on the main findings that show stable hepatic ECV values over time after contrast agent administration. ECV values at 5, 15, and 25 min after contrast injection were all equally suitable to detect hepatic fibrosis.

The calculation of the ECV was initially introduced for quantifying the myocardial extracellular fractional distribution volume [17] and was subsequently validated in histopathologic studies of myocardial fibrosis [9]. Although liver parenchyma with its dual blood supply, portal triad and parenchymal organisation has a more complex anatomy compared to the myocardium, hepatic ECV was also associated with severity fibrosis in the liver in an experimental animal model and correlated with different histopathologic parameters of liver fibrosis [10]. Furthermore, ECV mapping techniques have recently been adopted for liver imaging in humans. A proof-of-concept study has described ECV to be correlated with Sirius red staining in patients with diffuse liver disease [8]. Furthermore, bolus-only ECV measurements were independently correlated with different fibrosis stages in patients with chronic hepatitis B [18]. Also, ECV was shown to be superior to serum fibrosis indices in staging liver fibrosis in these patients [19]. Another study reported that hepatic ECV measured with equilibrium computed tomography (CT) imaging is associated with biopsy-derived collagen-proportionate area [2]. However, in comparison to MRI, the use of CT has some drawbacks, as it requires ionising radiation exposure and iodinated contrast agent administration, which is contraindicated in patients with reduced kidney function.

Beside that ECV has been validated against specific reference standards of disease severity in liver, heart and amyloid disease [2, 20, 21], recent data has shown that ECV was also strongly related to the fractional extracellular cellular volume of a three-dimensional engineered tissue model which was measured directly during its manufacture [22]. These results further support the principles underlying ECV estimation.

The calculation of ECV values is based on the assumption of a two-compartment model. After the application of an extracellular contrast agent, a steady state is achieved due to a rapid exchange with equal contrast concentrations between the blood and the extracellular space [16]. As ECV is calculated from the ratio of change in hepatic T1 relaxation time relative to blood-pool T1 after and before contrast administration, an adequate equilibrium is necessary to obtain valid results [23]. Several cardiac magnetic resonance studies focusing on myocardial ECV already discussed about whether equilibrium can only be achieved by a continuous contrast infusion technique or if a single bolus is sufficient to establish equilibrium within a certain time after contrast injection [9, 12, 24].

The results of this study showed that ECV assessment in the liver can be achieved using a bolus-only technique only, as measurements of ECV were quite constant over time. The data support the thesis of a dynamic equilibrium, which approximates the contrast equilibrium of a slow primed infusion of contrast media. Our results are concordant with previously published data on myocardial ECV. A cardiac magnetic resonance study, which compared bolus-only and primed infusion ECV assessment, reported that bolus-only ECV measurements are sufficient for ECV measurements across a range of cardiac disease. Both obtained ECV values correlated with histological collagen volume fraction [13]. Kawel et al. [23] described only slightly higher myocardial ECV values measured between 5 and 45 min. Miller et al. [25] described a linear increase of myocardial ECV over time, although the total mean difference between the measured ECV after 2 and 20 min was lower than 1.6%.

In our current study on liver ECV, the mean difference in hepatic ECV at 5 and 25 min in all three groups was lower than 2.1%. The slight increase was not statistically significant. There was also a strong linear relationship between hepatic ECV and Sirius red staining across a wide spectrum of liver fibrosis severity in this study. This strong relationship was independent of the time point of ECV assessment. Therefore, the results indicate that bolus-only hepatic ECV assessment is feasible. In practice, ECV value might likely be obtained at only one time point after contrast media injection.

Our study has several limitations. The T1 maps were acquired in a single transverse section and may therefore have missed more pronounced fibrotic disease that occurred in other planes. Also, the measurements were not corrected for hepatic steatosis or hepatic iron content because they are not expected in animal models. In humans, especially iron overload can influence quantitative measurements (*e.g.*, T1 relaxation times). However, when an iron-correction is applied, T1 values can grade hepatic fibrosis [26]. Finally, the results of this study have to be transferred to patients with chronic liver disease.

In conclusion, in this experimental animal study, we found that hepatic MRI-derived ECV using a bolus-only contrast injection technique is constant over time indicating a dynamic equilibrium. In clinical routine, post-contrast T1 relaxation times for ECV calculation might be obtained at only one time point during routine liver MRI, which might enhance and accelerate ECV assessment in the clinical workup of chronic liver disease. ECV values obtained at 5, 15, and 25 min after contrast administration were suitable to detect diffuse hepatic fibrosis.

## Abbreviations

CCl_4_: Carbon tetrachloride
ECV: Extracellular volume
MOLLI: Modified Look-Locker inversion-recovery
MRI: Magnetic resonance imaging
ROI: Region of interest

## References

[1] Blachier M, Leleu H, Peck-Radosavljevic M, Valla DC, Roudot-Thoraval F (2013). The burden of liver disease in Europe: a review of available epidemiological data. J Hepatol.

[2] Bandula S, Punwani S, Rosenberg WM (2015). Equilibrium contrast-enhanced CT imaging to evaluate hepatic fibrosis: initial validation by comparison with histopathologic sampling. Radiology.

[3] Ekstedt Mattias, Franzén Lennart E., Mathiesen Ulrik L., Thorelius Lars, Holmqvist Marika, Bodemar Göran, Kechagias Stergios (2006). Long-term follow-up of patients with NAFLD and elevated liver enzymes. Hepatology.

[4] Chalasani N, Younossi Z, Lavine JE (2012). The diagnosis and management of non-alcoholic fatty liver disease: practice guideline by the American Association for the Study of Liver Diseases, American College of Gastroenterology, and the American Gastroenterological Association. Hepatology.

[5] Poynard T, Lenaour G, Vaillant JC (2012). Liver biopsy analysis has a low level of performance for diagnosis of intermediate stages of fibrosis. Clin Gastroenterol Hepatol.

[6] Sandrin L, Fourquet B, Hasquenoph JM (2003). Transient elastography: a new noninvasive method for assessment of hepatic fibrosis. Ultrasound Med Biol.

[7] Arif-Tiwari H, Kalb B, Chundru S (2014). MRI of hepatocellular carcinoma: an update of current practices. Diagn Interv Radiol.

[8] Luetkens JA, Klein S, Traeber F (2018). Quantitative liver MRI including extracellular volume fraction for non-invasive quantification of liver fibrosis: a prospective proof-of-concept study. Gut.

[9] Flett AS, Hayward MP, Ashworth MT (2010). Equilibrium contrast cardiovascular magnetic resonance for the measurement of diffuse myocardial fibrosis: preliminary validation in humans. Circulation.

[10] Luetkens Julian A., Klein Sabine, Träber Frank, Schmeel Frederic C., Sprinkart Alois M., Kuetting Daniel L. R., Block Wolfgang, Uschner Frank E., Schierwagen Robert, Hittatiya Kanishka, Kristiansen Glen, Gieseke Juergen, Schild Hans H., Trebicka Jonel, Kukuk Guido M. (2018). Quantification of Liver Fibrosis at T1 and T2 Mapping with Extracellular Volume Fraction MRI: Preclinical Results. Radiology.

[11] Sharma P, Socolow J, Patel S, Pettigrew RI, Oshinski JN (2006). Effect of Gd-DTPA-BMA on blood and myocardial T1 at 1.5T and 3T in humans. J Magn Reson Imaging.

[12] Schelbert EB, Testa SM, Meier CG (2011). Myocardial extravascular extracellular volume fraction measurement by gadolinium cardiovascular magnetic resonance in humans: slow infusion versus bolus. J Cardiovasc Magn Reson.

[13] White SK, Sado DM, Fontana M (2013). T1 mapping for myocardial extracellular volume measurement by CMR: bolus only versus primed infusion technique. JACC Cardiovasc Imaging.

[14] Trebicka Jonel, Hennenberg Martin, Schulze Pröbsting Andrea, Laleman Wim, Klein Sabine, Granzow Michaela, Nevens Frederik, Zaagsma Johan, Heller Jörg, Sauerbruch Tilman (2009). Role of β3-adrenoceptors for intrahepatic resistance and portal hypertension in liver cirrhosis. Hepatology.

[15] Trebicka J, Hennenberg M, Odenthal M (2010). Atorvastatin attenuates hepatic fibrosis in rats after bile duct ligation via decreased turnover of hepatic stellate cells. J Hepatol.

[16] Schelbert EB, Messroghli DR (2016). State of the art: clinical applications of cardiac T1 mapping. Radiology.

[17] Arheden H, Saeed M, Higgins CB (1999). Measurement of the distribution volume of gadopentetate dimeglumine at echo-planar MR imaging to quantify myocardial infarction: comparison with 99mTc-DTPA autoradiography in rats. Radiology.

[18] Jin K, Wang H, Zeng M (2019). A comparative study of MR extracellular volume fraction measurement and two-dimensional shear-wave elastography in assessment of liver fibrosis with chronic hepatitis B. Abdom Radiol (NY).

[19] Wang HQ, Jin KP, Zeng MS (2019). Assessing liver fibrosis in chronic hepatitis B using MR extracellular volume measurements: comparison with serum fibrosis indices. Magn Reson Imaging.

[20] Bandula S, Banypersad SM, Sado D (2013). Measurement of tissue interstitial volume in healthy patients and those with amyloidosis with equilibrium contrast-enhanced MR imaging. Radiology.

[21] de Meester de Ravenstein C, Bouzin C, Lazam S et al (2015) Histological validation of measurement of diffuse interstitial myocardial fibrosis by myocardial extravascular volume fraction from modified look-locker imaging (MOLLI) T1 mapping at 3 T. J Cardiovasc Magn Reson 17:48. 10.1186/s12968-015-0150-0 10.1186/s12968-015-0150-0 PMC4464705 26062931

[22] Bandula S, Magdeldin T, Stevens N (2016). Initial validation of equilibrium contrast imaging for extracellular volume quantification using a three-dimensional engineered tissue model. J Magn Reson Imaging.

[23] Kawel N, Nacif M, Zavodni A (2012). T1 mapping of the myocardium: intra-individual assessment of post-contrast T1 time evolution and extracellular volume fraction at 3T for Gd-DTPA and Gd-BOPTA. J Cardiovasc Magn Reson.

[24] Thornhill RE, Prato FS, Wisenberg G, White JA, Nowell J, Sauer A (2006). Feasibility of the single-bolus strategy for measuring the partition coefficient of Gd-DTPA in patients with myocardial infarction: independence of image delay time and maturity of scar. Magn Reson Med.

[25] Miller CA, Naish JH, Bishop P (2013). Comprehensive validation of cardiovascular magnetic resonance techniques for the assessment of myocardial extracellular volume. Circ Cardiovasc Imaging.

[26] Banerjee R, Pavlides M, Tunnicliffe EM (2014). Multiparametric magnetic resonance for the non-invasive diagnosis of liver disease. J Hepatol.

